# Allogeneic hematopoietic stem cell transplantation for acute leukemia with Gilbert's syndrome

**DOI:** 10.1186/1756-8722-4-9

**Published:** 2011-03-09

**Authors:** Guo-Pan Yu, Qian-Li Jiang, Zhi-Ping Fan, Jie Zhao, Qi Wei, Jing Sun, Fan-Yi Meng, Qi-Fa Liu

**Affiliations:** 1 Department of Hematology, Nanfang Hospital, Southern Medical University, Guangdong, China

## Abstract

Acute leukemia with coexisting Gilbert's syndrome treated by allogeneic hematopoietic stem cell transplantation (allo-HSCT) is rarely reported. Here we described a case whose transaminase levels were almost normal, although transient hyperbilirubinemia repeatedly happened during chemotherapy.

## 

To the editor:

We have seen a 52-year-old man with AML-FAB M2a subtype, who had no history of viral hepatitis. He had history of mild indirect hyperbilirubinemia with normal transaminase levels after he took paracetamols in the past two years, and the same phenomenon occurred to his siblings, children and nephews. He received three cycles of chemotherapy containing daunorubicin, idarubicin, pirarubicin, cytarabine, and obtained CR in the first cycle. His bilirubin level was normal before chemotherapy; however, mild non-hemolytic indirect hyperbilirubinemia happened to him during each cycle of chemotherapy (Figure 1). Neither autoimmune antibodies, nor serology of viral hepatitis were positive. CT scan revealed that liver parenchyma, gallbladder and bile duct were all normal. The histopathology of a liver biopsy showed mild chronic hepatitis and hepatocellular cholestasis. The expression of UGT1A1 gene was found to decrease by 20%. The diagnosis of Gilbert's syndrome was confirmed.

**Figure 1:**
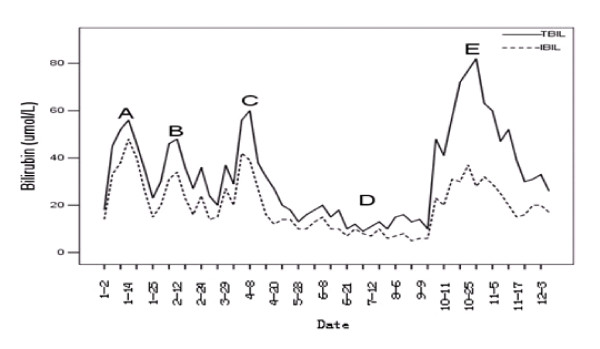
**Bilirubin level during chemotherapy and allogeneic hematopoietic stem cell transplantation**. Chronological dates of the treatment courses were indicated. (A). the first cycle of chemotherapy of DA regimen [Daunorubicin 60 mg/d from day 1 to 3, Cytarabine (Ara-C) 200 mg/d from day 1 to 7]. (B). the second cycle of chemotherapy of IA (Idarubicin 10 mg/d from day 1 to 3, Ara-C 200 mg/d from day 1 to 7). (C). the third cycle of chemotherapy of TA regimen (Pirarubicin 40 mg/d from day 1 to 3, Ara-C 200 mg/d from day 1 to 7). (D). allo-HSCT [fludarabine (30 mg/m2/d, on days -6 to -2) and busulfan (3.2 mg/kg/d, on days -6 to -3) for conditioning; Phenytoin for prophylaxis of busulfan's side affect. Tacrolimus; methotrexate and mycophenolate mofetil (0.5 g twice a day on days 0 to +28) for GVHD prophylaxis; Caspofungin for prophylaxis of fungal infections]. Tropisetron was used during the chemotherapy and conditioning. (E). period of acute GVHD [alanine aminotransferase 321 U/L, aspartate aminotransferase 284 U/L, total bilirubin 61.7 umol/L (3.6 mg/dL), and indirect bilirubin 34.1 umol/L (2.0 mg/dL)].

After 3 cycles of chemotherapy, the patient received transplantation from his HLA-identical sibling sister who was also diagnosed to have GS. The conditioning regimen included fludarabine and busulfan. Hematopoietic engraftment was observed on day +11. In the absence of GVHD, the levels of transaminase and bilirubin were almost normal within 100 days post-transplantation (Figure 1). The patient received G-CSF mobilized donor peripheral lymphocyte transfusion on days + 60 and +90, respectively. He developed skin rash and abnormal liver function on day +123 (Figure 1), consistent with grade II acute GVHD. After one month treatment with Methylprednisolone, tacrolimus and methotrexate, his liver dysfunction gradually returned to normal. At the last follow-up on day +510, his liver function was still normal, and he remained leukemia free.

GS is a common condition; its prevalence has been described in 3-10% of the general population [1]. With mild symptoms and reversible indirect hyperbilirubinemia, most cases of GS remain undiagnosed and they may become evident after exercise, stress, drug administration, prolonged fasting or inter-current diseases [2]. Several drugs have been reported to induce hyperbilirubinemia to GS patients, such as paracetamol, nilotinib, and pazopanib [3,4]. In our case, hyperbilirubinemia happened to the patient after each cycle of the chemotherapy, but it did not occur during the conditioning and within 100 days post-transplantation. This suggests that anthracyclines and/or cytarabine might be an inhibitor of UGT1A1 enzyme.

Some reports suggest that GS might be a risk factor of cancer [5,6]. GS with coexisting hematological malignancies including AML has been reported in individuals, but the incidence of hematological malignancy in GS is still unknown [1]. Among these cases, just like ours, non-hemolytic low-grade indirect hyperbilirubinemia after chemotherapy occurred repeatedly, but it did not effect the treatment of hematologic malignancies. In clinical practice, hyperbilirubinemia is suggestive of hemolysis, drug-induced liver damage or other conditions, but GS is not very frequently considered [2]. In our case, we initially attributed hyperbilirubinemia to drug-induced liver damage in the first two cycles of chemotherapy.

To our knowledge, there is little description of the onset of hyperbilirubinemia in GS patients who received HSCT. Ruiz-Arguelles GJ, et al. [2] reported seven cases of acute leukemia with coexisting GS, of which 3 patients accepted chemotherapy alone, 3 cases treated by allo-HSCT and one received auto-PBSCT. All seven patients developed indirect hyperbilirubinemia during the course of their treatment. No patients died from the liver dysfunction. In our case, transaminase and bilirubin levels were almost normal during the conditioning and within 100 days after transplantation, although indirect hyperbilirubinemia repeatedly happened during chemotherapy with daunorubicin, idarubicin, and cytarabine prior to HSCT. This may suggest that fludarabine and busulfan had low liver toxicity in GS patients [7]. It could also be possible that phenytoin, an inducer of UGT1A1 activity used during the conditioning, prevented hyperbilirubinemia [8]. It has been reported that UGT1A1 inducers, including phenobarbital, rifampin and phenytoin, were used in the diagnosis and treatment of GS [8,9].

## List of abbreviations

GS: Gilbert's syndrome; allo-HSCT: Allogeneic hematopoietic stem cell transplantation; AML: acute myeloid leukemia; CR: complete remission; CT: Computed tomography; UGT1A1: uridine 5'-diphosphoglucose glucuronosyl transferase; HLA: human leukocyte antigen; GVHD: graft-versus-host disease; G-CSF: granulocyte-colony-stimulating factor; PCP: pneumocystis carinii pneumonia.

## Competing interests

The authors declare that they have no competing interests.

## Authors' contributions

All authors were involved in the provision of clinical care of the patient and the collection of data and the review of the manuscript. GPY: supplied the acquisition of data, analysis and interpretation of data, drafting of manuscript; QFL: provided the conception and design of the paper, revised it critically for important intellectual content, and final approval of the version to be submitted.

## Authors' information

QFL, MD, Ph.D, Department of Hematology, Nanfang Hospital, Southern Medical University; Member of the Asian-Pacific Society of Hematology; Member of Blood Branch of the Chinese Medical Association; Member of the Chinese hematopoietic stem cell transplant group; Standing Committee member of Chinese Anti-Cancer Association of Professional Committee of Hematology Branch; editor of more than 10 journals.
